# Evolutionary Insights Into Two Widespread Ectomycorrhizal Fungi (*Pisolithus*) From Comparative Analysis of Mitochondrial Genomes

**DOI:** 10.3389/fmicb.2021.583129

**Published:** 2021-07-05

**Authors:** Peng Wu, Tian Yao, Yuanhang Ren, Jinghua Ye, Yuan Qing, Qiang Li, Mingying Gui

**Affiliations:** ^1^Yunnan Plateau Characteristic Agricultural Industry Research Institute, Yunnan Agricultural University, Kunming, China; ^2^School of Food and Biological Engineering, Chengdu University, Chengdu, China; ^3^College of Information Science and Engineering, Chengdu University, Chengdu, China; ^4^Panxi Featured Crops Research and Utilization Key Laboratory of Sichuan Province, Xichang University, Xichang, China

**Keywords:** mitochondrial genome, plasmid, intron, gene rearrangement, evolution, phylogenetic analysis

## Abstract

The genus *Pisolithus* is a group of global ectomycorrhizal fungi. The characterizations of *Pisolithus* mitochondrial genomes have still been unknown. In the present study, the complete mitogenomes of two *Pisolithus* species, *Pisolithus microcarpus*, and *Pisolithus tinctorius*, were assembled and compared with other Boletales mitogenomes. Both *Pisolithus* mitogenomes comprised circular DNA molecules with sizes of 43,990 and 44,054 bp, respectively. Comparative mitogenomic analysis showed that the *rps3* gene differentiated greatly between Boletales species, and this gene may be subjected to strong pressure of positive selection between some Boletales species. Several plasmid-derived genes and genes with unknown functions were detected in the two *Pisolithus* mitogenomes, which needs further analysis. The two *Pisolithus* species show a high degree of collinearity, which may represent the gene arrangement of the ancestors of ectomycorrhizal Boletales species. Frequent intron loss/gain events were detected in Boletales and basidiomycetes, and intron P717 was only detected in *P. tinctorius* out of the eight Boletales mitogenomes tested. We reconstructed phylogeny of 79 basidiomycetes based on combined mitochondrial gene dataset, and obtained well-supported phylogenetic topologies. This study served as the first report on the mitogenomes of the family Pisolithaceae, which will promote the understanding of the evolution of *Pisolithus* species.

## Introduction

*Pisolithus* species are ectomycorrhizal fungi with wide geographic distribution and host ranges, such as pine, oak and so on (Marx, 1977; Hitchcock et al., 2011). The symbiosis of *Pisolithus* species with trees promotes the carbon and nutrient cycling of the forest ecosystem (Cairney et al., 1989). In addition, the *Pisolithus* symbionts can promote the growth of plants, the absorption of mineral elements, and enhance the ability of plants to cope with abiotic stress, including drought, heavy metals and nutritional deficiencies (Jourand et al., 2014; Shi et al., 2017; Sebastiana et al., 2018). Up to now, more than ten species have been described in the genus *Pisolithus*. The limited morphological characteristics caused confusion in the classification of subspecies, varieties and species complex in the genus *Pisolithus* (Junghans et al., 1998; Hitchcock et al., 2003). The mitochondrial genome is a powerful tool to analyze the phylogenetic relationship of basidiomycetes (Basse, 2010). Up to now, six mitogenomes from the order Boletales have been reported, including four ectomycorrhizal Boletales species, *Rhizopogon salebrosus*, *R. vinicolor* (Li et al., 2019a), *Paxillus involutus*, *P. rubicundulus* (Li et al., 2020b), and two saprophytic Boletales species, *Coniophora olivacea* (MT375015), and *C. puteana* (MT375016). However, no complete mitogenome of *Pisolithus* species has been published.

Mitochondria play an important role in environmental adaptation, stress regulation and growth of eukaryotes (Ernster and Schatz, 1981). As the energy factory of cells, the genome of mitochondria mainly encodes respiratory chain related proteins, components of the ATPase and genes required for translation, such as rRNA and tRNA genes (McBride et al., 2006). Mutations in the mitogenome could lead to aging, disease, and even death of eukaryotic species (Ernster and Schatz, 1981; McBride et al., 2006; Gu et al., 2019). The mitogenome of basidiomycetes varies greatly in gene arrangement, genome size, intron type and number, repeat sequence content, and gene composition (Li et al., 2018d, 2020a; Sandor et al., 2018). Some of these characteristics can be used to infer the phylogenetic relationship of basidiomycetes and understand the origin or evolution of basidiomycetes (Richards et al., 1998; Li et al., 2015). In addition, the mitogenome of basidiomycetes contains several available molecular markers, which are widely used in phylogeny (Richards et al., 1998; Li et al., 2019c). So far, several nuclear genes, such as the internal transcribed spacer (ITS), the second largest subunit of RNA polymerase II (*RPB2*), beta-tubulin sequences, and translation elongation factor 1-α (*tef1-*α), have been widely used in the taxonomic classification and phylogeny of basidiomycetes (James et al., 2006). With more and more fungal nuclear genomes sequenced, the construction of fungal phylogeny based on whole genome sequence has become a trend (Li et al., 2018a). Mitogenome provides more genetic information than single nuclear gene molecular markers, and is easier to be obtained than the whole nuclear genome. So the mitogenome has become an effective supplementary tool to analyze fungal phylogeny. Compared with the mitogenome of animals, the mitogenome of fungi contains introns, and their types and numbers vary greatly among different fungal species (Kolesnikova et al., 2019; Li et al., 2020c). The number of available fungal mitogenomes, particularly basidiomycete mitogenomes, is far less than that of animals, or even less than the available nuclear genomes of fungi^1^. So far, two *Pisolithus* nuclear genomes have been reported, including *Pisolithus microcarpus*, and *Pisolithus tinctorius* (Kohler et al., 2015), but their mitogenomes have not been resolved.

In the present study, mitogenomes of two *Pisolithus* species, including *P. microcarpus* and *P. tinctorius*, were assembled and compared with other Boletales mitogenomes to assess the variations and conservation based on their gene arrangement, genome size, gene content and gene structure. Phylogenetic relationships of 79 basidiomycetes were revealed based on the combined mitochondrial datasets. Intron dynamics in *cox1* genes of 79 basidiomycetes were also detected to understand the origin and evolution of introns in the phylum Basidiomycota. This study served as the first report on mitogenomes of the genus *Pisolithus* and the family Pisolithaceae, which will promote the understanding of the phylogeny, evolution and population genetics of *Pisolithus* species.

## Materials and Methods

### *Pisolithus* Mitogenomes Assembly and Annotation

The raw sequencing data of *P. microcarpus* and *P. tinctorius* used for mitogenomes assembly were downloaded from the Sequence Read Archive (SRA) database under the accession numbers of SRR3927183 and SRR3927210, respectively, which were submitted by the DOE Joint Genome Institute (Kohler et al., 2015). We conducted several quality control steps to generate clean data from the raw sequencing data, including removing adapter reads by ngsShoRT (Chen et al., 2014) and filtering reads with low-quality values by AdapterRemoval v 2 (Schubert et al., 2016). The two *Pisolithus* mitogenomes were assembled using the SPAdes 3.9.0 software based on the obtained clean reads (Bankevich et al., 2012). Gaps between contigs were filled using the MITObim V1.9 software (Hahn et al., 2013). The complete mitogenomes of *P. microcarpus* and *P. tinctorius* were obtained and further annotated according to the previously described methods (Li et al., 2018b). The protein-coding genes (PCGs), open reading frames (ORFs), introns, rRNA genes, and tRNA genes were first annotated by MFannot (Valach et al., 2014) and MITOS (Bernt et al., 2013). Then the annotated ORFs were verified or modified using the NCBI Open Reading Frame Finder (Coordinators, 2017), and further annotated by BLASTP searches against the NCBI non-redundant protein sequence database (Bleasby and Wootton, 1990). Intron-exon borders of PCGs were determined by the exonerate v2.2 software (Slater and Birney, 2005). tRNA genes in the two *Pisolithus* mitogenomes were also predicted using tRNAscan-SE v1.3.1 (Lowe and Chan, 2016). The graphical maps of the two *Pisolithus* mitogenomes were drawn using OGDraw v1.2 (Lohse et al., 2013).

### Sequence Analysis of *Pisolithus* Mitogenomes

We calculated base compositions of the two *Pisolithus* mitogenomes and six Boletales mitogenomes using the DNASTAR Lasergene v7.1^2^. Strand asymmetries of all the eight Boletales mitogenomes were assessed using the following formulas: AT skew = [A − T]/[A + T], and GC skew = [G − C]/[G + C]. Pairwise genetic distances between each pair of the 15 core PCGs (*atp6, atp8, atp9, cob, cox1, cox2, cox3, nad1, nad2, nad3, nad4, nad4L, nad5, nad6*, and *rps3*) in the eight Boletales mitogenomes tested were calculated using MEGA v6.06 (Caspermeyer, 2016) based on the Kimura-2-parameter (K2P) substitution model. We also detected the synonymous substitution rates (*Ks*) and non-synonymous substitution rates (*Ka*) for core PCGs in the eight Boletales mitogenomes using the DnaSP v6.10.01 software (Rozas et al., 2017). Gene collinearity analysis of the eight Boletales species was conducted using Mauve v2.4.0 software (Darling et al., 2004).

### Repetitive Element Analysis

We conducted BLASTN searches (Chen et al., 2015) of the two *Pisolithus* mitogenomes against themselves to detect any interspersed repeats or intra-genomic duplications in the two *Pisolithus* mitogenomes, based on an *E*-value of <10^–10^. Tandem repeats (>10 bp in length) in the two *Pisolithus* mitogenomes were detected using the Tandem Repeats Finder (Benson, 1999). We also performed BlastN searches of the two *Pisolithus* mitogenomes against their published nuclear genomes (GCA_000827275.1; GCA_000827335.1) to detect if there were any gene fragments that natural transferred between the nuclear and mitochondrial genomes of the two *Pisolithus* species.

### Comparative Mitogenomic Analysis and Intron Analysis

In the present study, we compared the genome sizes, gene numbers, intron numbers, base compositions, and gene arrangements of the eight Boletales mitogenomes reported to assess variations and conservativeness between different Boletales species. We assigned introns of *cox1* genes in 79 published Basidiomycota mitogenomes into different position classes (Pcls) according to the method described by Ferandon et al. (2010). We first aligned the *cox1* genes of 79 Basidiomycota mitogenomes with the reference *cox1* gene of the medical fungus *G. calidophilum* (Li et al., 2019c) using Clustal W (Thompson et al., 1994). Introns were named according to the insert sites (nt) in corresponding reference gene. Each Pcl was constituted by introns inserted at the same position of corresponding reference *cox1* gene (Cheng et al., 2021). Pcls with the same insert sites were considered as orthologous introns and had high sequence similarities (Ferandon et al., 2010).

### Phylogenetic Analysis

In order to investigate the phylogenetic relationships of Boletales species and other Basidiomycota species, we constructed a phylogenetic tree of 79 species based on the combined mitochondrial gene datasets (15 core PCGs) (Li et al., 2021b). The mitogenome of *Annulohypoxylon stygium* from the phylum Ascomycota was used as an outgroup (Deng et al., 2018). We first aligned individual mitochondrial genes using MAFFT v7.037 software (Katoh et al., 2019), and then concatenated them into a combined mitochondrial gene dataset using SequenceMatrix v1.7.8 (Vaidya et al., 2011). We conducted partition homogeneity test to detect if there was potential phylogenetic conflict between different mitochondrial genes. PartitionFinder v2.1.1 software (Lanfear et al., 2017) was used to determine partitioning schemes for the mitochondrial gene set and best-fit models for phylogeny. The phylogenetic analysis was conducted using both maximum likelihood (ML) and bayesian inference (BI) methods (Li et al., 2018c). We used RAxML v 8.0.0 (Stamatakis, 2014) to conduct the ML analysis, and used MrBayes v3.2.6 (Ronquist et al., 2012) to perform the BI analysis. When conducted the ML analysis, bootstrap values (BS) were assessed through an ultrafast bootstrap approach with 10,000 replicates. BI analysis was conducted according to the previously described methods (Wang et al., 2020; Ye et al., 2020).

### Data Availability

The complete mitogenomes of *P. microcarpus* and *P. tinctorius* were deposited in the GenBank database under the accession numbers of MT577034 and MT577035, respectively.

## Results

### Characterization and PCGs of *Pisolithus* Mitogenomes

**FIGURE 1:**
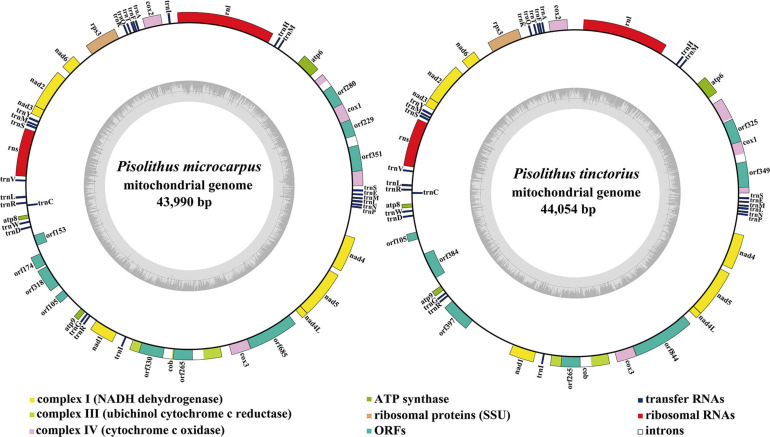
Circular maps of the two *Pisolithus* mitogenomes. Genes are represented by different colored blocks. Colored blocks outside each ring indicate that the genes are on the direct strand, while colored blocks within the ring indicates that the genes are located on the reverse strand. The inner graph is the GC content of mitochondrial sequences, and the circle inside the GC content graph marks the 50% threshold.

The mitogenomes of *P. microcarpus* and *P. tinctorius* were all circular, with sizes of 43,990 and 44,054 bp, respectively (Figure 1). The GC contents of the *P. microcarpus* and *P. tinctorius* mitogenomes were 23.34 and 22.18%, respectively (Supplementary Table 1). Both the two *Pisolithus* species had negative AT skews and positive GC skews. There were 25 and 22 PCGs present in the *P. microcarpus* and *P. tinctorius* mitogenomes, respectively. Both the two mitogenomes contained a set of core PCGs, including *atp6*, *atp8*, *atp9*, *cob*, *cox1*, *cox2*, *cox3*, *nad1*, *nad2*, *nad3*, *nad4*, *nad4L*, *nad5*, *nad6*, and *rps3* (Supplementary Table 2). In addition, we detected two plasmid-derived genes in the mitogenome of *P. tinctorius*, which encoded DNA polymerase and RNA polymerase. We also detected five and two PCGs with unknown functions in the *P. microcarpus* and *P. tinctorius* mitogenomes, respectively. Four and three introns were detected in the *P. microcarpus* and *P. tinctorius* mitogenomes, respectively, which were distributed in *cox1* and *cob* genes of *Pisolithus* mitogenomes. Five and three intronic ORFs were detected in the *P. microcarpus* and *P. tinctorius* mitogenomes, respectively, which encoded two families of homing endonucleases, namely GIY YIG and LAGLIDADG endonucleases. The first intron of *cox1* gene in *P. microcarpus* contained two intronic ORFs.

### RNA Genes in *Pisolithus* Mitogenomes

Both the *P. microcarpus* and *P. tinctorius* mitogenomes contained two rRNA genes, namely the small subunit ribosomal RNA gene (*rns*) and the large subunit ribosomal RNA gene (*rnl*) (Supplementary Table 2). The average lengths of *rns* and *rnl* genes in the two *Pisolithus* mitogenomes were 1,973 and 3,768 bp, respectively. The mitogenome of *P. microcarpus* contained shorter *rns* and longer *rnl* genes than that of *P. tinctorius*.

**FIGURE 2:**
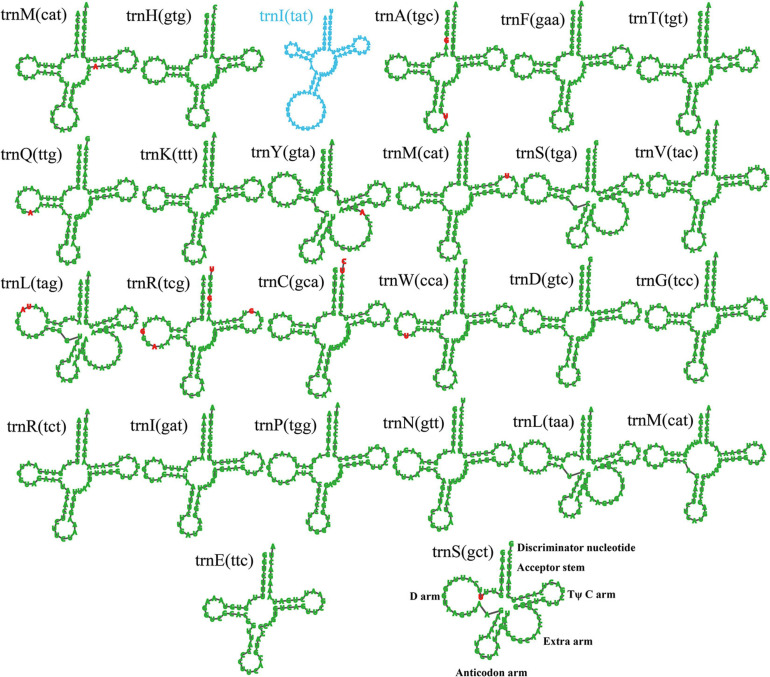
Putative secondary structures of tRNA genes in the two *Pisolithus* mitogenomes. tRNA genes with green color represent tRNAs shared by the two *Pisolithus* species, while tRNA genes with blue color represent the unique tRNAs in *Pisolithus microcarpus*. Residues conserved across the two mitogenomes are shown in green, while variable sites are shown in red. All genes are shown in order of occurrence in the mitogenome of *Pisolithus microcarpus*, starting from *trnM*.

We detected 26 and 25 tRNA genes in the mitogenomes of *P. microcarpus* and *P. tinctorius*, respectively (Supplementary Table 2). The mitogenome of *P. microcarpus* contained an additional *trnI* compared with the *P. tinctorius* mitogenome. All tRNAs detected in the two *Pisolithus* mitogenomes were folded into classical cloverleaf structures (Figure 2). The length of these tRNAs ranged from 71 bp to 86 bp, and the *trnS* gene was the largest. The extra stem/loop regions were found to be the main factors contributing to size variations of tRNA genes in *Pisolithus*. Ten of the 26 tRNAs shared in the two *Pisolithus* mitogenomes contained sites that varied between the two *Pisolithus* mitogenomes. We detected a total of 15 variable sites between the two *Pisolithus* mitogenomes, of which seven occurred on the D arm.

### Repeats of *Pisolithus* itogenomes

A total of two and four intra-genomic duplications were detected in the mitogenomes of *P. microcarpus* and *P. tinctorius*, respectively (Supplementary Table 3). The size of the four intra-genomic duplications ranged from 39 to 52 bp, and the pair-wise nucleotide similarities were between 79.49 and 100%. The largest intra-genomic duplications were observed in the coding regions of intronic ORFs, orf349 and orf325, in the *P. tinctorius* mitogenome. Intra-genomic duplications in the *P. microcarpus* mitogenomes were found located in the protein coding regions of *atp6*, as well as in the intergenic region between neighboring genes *atp6* and *trnM*. Intra-genomic duplications accounted for 0.23 and 0.41% of the *P. microcarpus* and *P. tinctorius* mitogenomes, repectively. A total of 14 and 36 tandem repeats were detected in the mitogenomes of *P. microcarpus* and *P. tinctorius*, repectively, (Supplementary Table 4). The largest tandem repeat was found located in the intergenic region between *nad6* and *nad2* in the *P. tinctorius* mitogenome, with a size of 144 bp. Individual tandem repeat in the two *Pisolithus* mitogenomes contained 2 to 33 copies, with the most copy number observed in the *P. tinctorius* mitogenome. Tandem repeats accounted for 1.58 and 4.96% of the *P. microcarpus* and *P. tinctorius* mitogenomes, repectively.

We conducted BlastN searches of the two *Pisolithus* mitogenomes against their published nuclear genomes to detect any gene fragments that transferred between the mitochondrial and nuclear genomes. There were 4 and 22 aligned fragments found between mitochondrial and nuclear genomes of *P. microcarpus* and *P. tinctorius*, respectively (Supplementary Table 5). The length of these repetitive fragments ranged from 31 to 7,709 bp, and the pair-wise nucleotide similarities were between 93.10 and 100%. The largest aligned fragment was found located between *rnl* and *trnM* genes in the mitogenome of *P. tinctorius*. A total of 129 and 36,944 bp aligned fragments were detected in the mitogenomes of *P. microcarpus* and *P. tinctorius*, respectively. Large aligned fragments found between mitochondrial and nuclear genomes of the two *Pisolithus* species indicated that gene fragment transfer events may have occurred in the evolution of *Pisolithus* species.

### Mitochondrial Gene Arrangement in Boletales Species

**FIGURE 3:**
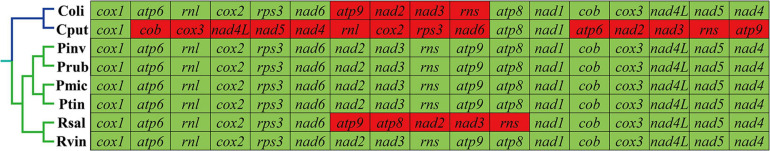
Mitochondrial gene arrangement analyses of eight Boletales species. All genes are shown in order of occurrence in the mitochondrial genome, starting from *cox1*. Fifteen core protein coding genes and two rRNA genes were included in the gene arrangement analysis. Genes with a green background indicate that they are conservative in Boletales species. The genes with red background indicate that they have gene rearrangements. The phylogenetic positions of the eight Boletales species were established using the Bayesian inference (BI) method and Maximum Likelihood (ML) method based on concatenated mitochondrial genes. Coli, *C. olivacea*; Cput, *C. puteana*; Pinv, *P. involutus*; Prub, *P. rubicundulus*; Pmic, *P. microcarpus*; Ptin, *P. tinctorius*; Rsal, *R. salebrosus*; Rvin, *R. vinicolor*.

To date, six complete mitogenomes from the order Boletales have been reported (Li et al., 2019a). In the present study, we compared gene arrangements of 15 core PCGs and 2 rRNA genes in the eight Boletales species. Five out of the eight Boletales species had identical gene arrangements, including *Rhizopogon vinicolor*, *Paxillus involutus*, *Paxillus rubicundulus*, *P. microcarpus*, and *P. tinctorius*, which may represent the gene arrangement of the common ancestor of ectomycorrhizal Boletales species (Figure 3). However, we detected large-scale gene rearrangements in the other three Boletales species, including *C. olivacea*, *Coniophora puteana*, and *Rhizopogon salebrosus*. Compared with the gene arrangement of the putative ancestor of ectomycorrhizal Boletales, the three Boletales species were found underwent large-scale gene rearrangements, involving gene migrations, insertions, and inversions.

**FIGURE 4:**
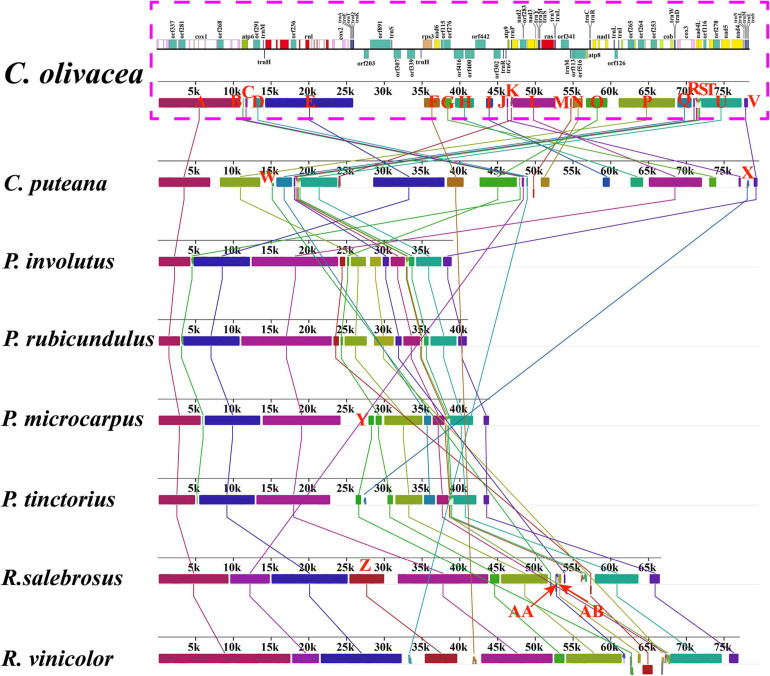
Gene collinearity analysis of eight Boletales species using Mauve v2.4.0. Color blocks of the same color represent homologous regions between different mitogenomes, which are represented by different capital letters. The schematic diagram of *Coniophora olivacea’s* mitogenome is shown at the top of the picture.

A total of 28 homologous regions were detected between the 8 Boletales species using Mauve (Figure 4). The mitogenomes of *P. microcarpus*, *P. tinctorius*, and *R. vinicolor* were found containing the homologous region Y, which have never been detected in other Boletales species. The Y region contained some intergenetic sequences, which may be obtained from the same ancestor. Homologous region X was unique homologous region in *C. puteana* and *P. tinctorius* mitogenomes. The two *Coniophora* contained unique homologous regions G, H, I, J, K, M, and N, which were involved in *orf276*, *orf113*, and *orf516* encoding DNA polymerases, and *orf416*, *orf400*, and *orf302* encoding proteins with unknown functions. The results showed that the saprophytic Boletales species (*Coniophora*) differentiated from ectomycorrhizal Boletales species in gene content. Collinearity analysis indicated that *Pisolithus*, *Paxillus*, and *Rhizopogon* species showed a high degree of collinearity, while large-scale gene rearrangements were found between *Coniophora* species, indicating gene arrangement differentiated in *Coniophora* species.

### Evolution of Core PCGs in Boletales

**FIGURE 5:**
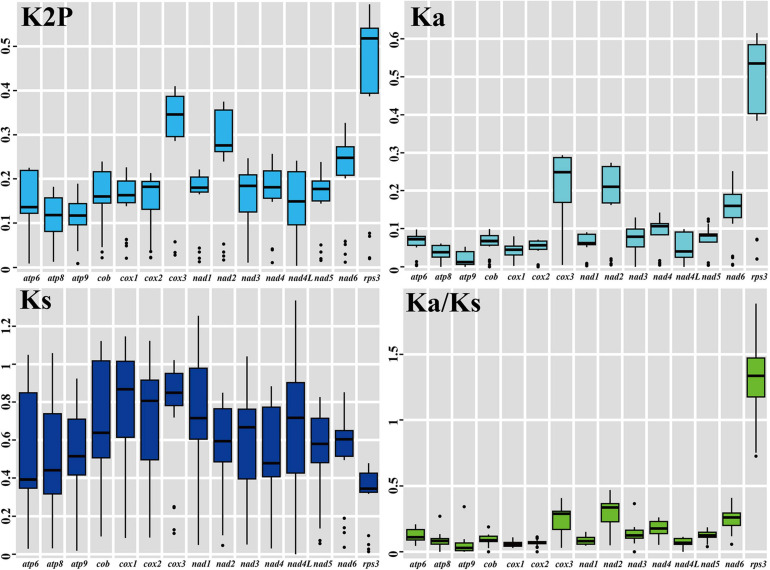
Genetic analysis of 15 core protein coding genes conserved in the 8 Boletales mitogenomes. K2P, pairwise genetic distances between each pair of the 15 core PCGs in the 8 Boletales mitogenomes based on the Kimura-2-parameter model. *Ka*, non-synonymous substitution site; *Ks*, synonymous substitution site. Species and NCBI accession number used for genetic analysis in the present study are listed in Supplementary Table 6.

The largest K2P genetic distance was found in *rps3* gene among the 15 core PCGs detected, which indicted the *rps3* gene varied greatly between the 8 Boletales species (Figure 5). Large gene differentiations were also observed in the *cox3* and *nad2* genes between the 8 Boletales species tested. The lowest mean K2P genetic distances were observed in the *atp8* and *atp9* genes among the 15 core PCGs, indicating that the two genes were highly conserved between the 8 Boletales species.

The highest mean non-synonymous substitution rate (*Ka*) was observed in the *rps3* gene among the 15 core PCGs detected, followed by the *cox3* gene (Figure 5), while the lowest mean *Ka* value was observed in the *atp9* gene among the 15 core PCGs from the eight Boletales species. The *cox1* genes in the 8 Boletales species were found exhibited the highest mean synonymous substitution rate (*Ks*), while the *rps3* gene exhibited the lowest mean *Ks* value among the 15 PCGs detected. We found the *Ka/Ks* values for 14 of the 15 core PCGs were less than 1, indicating that the 14 core genes were subjected to purifying selection in evolution. However, we found the *Ka/Ks* value of the *rps3* was >1 between some Boletales species, including between *P. microcarpus* and *Paxillus involutus*, between *P. microcarpus* and *Paxillus rubicundulus*, between *P. tinctorius* and *C. olivacea*, between *P. tinctorius* and *C. puteana*, as well as between *Rhizopogon vinicolor* and *R. salebrosus*, indicating that the *rps3* gene may be under strong positive selection pressure in some Boletales species.

### Intron Dynamics in *cox1* Genes of Basidiomycota Species

Intron dynamics in *cox1* genes of 79 Basidiomycota species were analyzed. Species included in the intron analysis accounted for over 2/3 of Basidiomycota mitogenomes available in the NCBI database. We detected 1,071 introns in the 79 Basidiomycota species, and most of them belonged to the group I. The number of intron in individual Basidiomycota species ranged from 0 to 46, and the *cox1* gene was found containing the largest number of introns among all the host genes detected in Basidiomycota. A total of 488 introns were found located in *cox1* genes of the 79 Basidiomycota species, which occupied 45.56% of the introns found in the mitogenomes of the 79 examined basidiomycetes.

**FIGURE 6:**
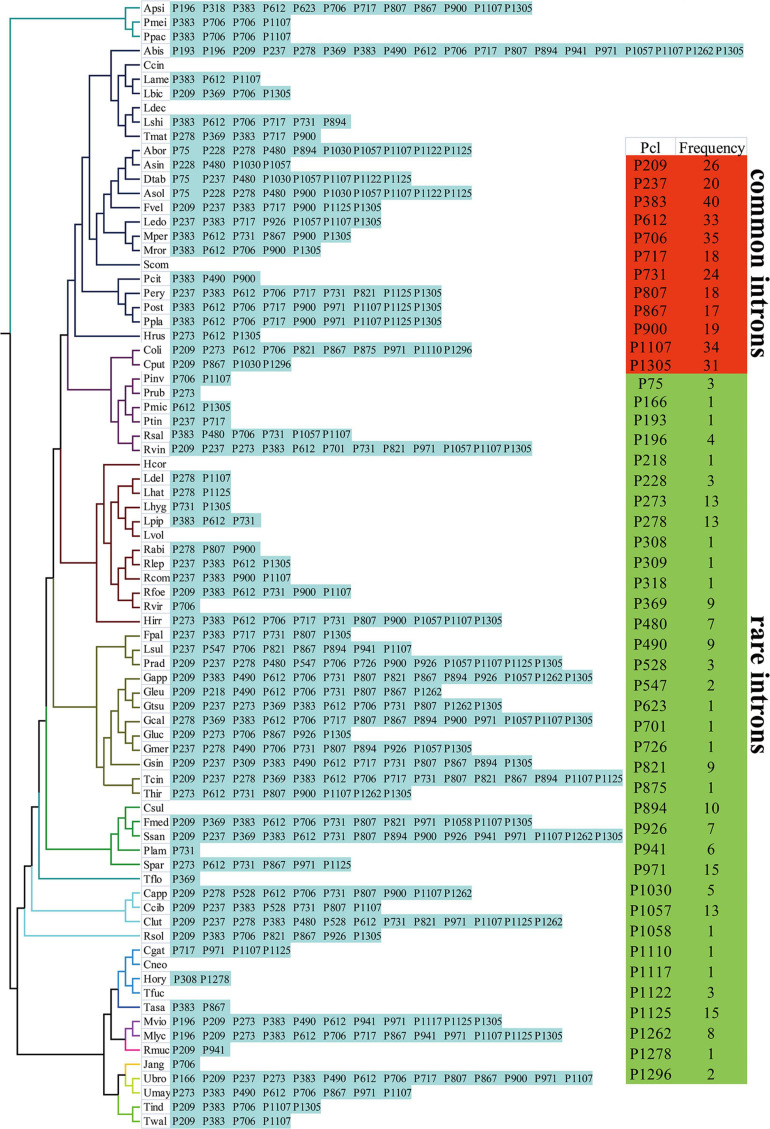
Position class (Pcl) information of *cox1* genes in the 79 Basidiomycota species. Introns in *cox1* genes of 79 published Basidiomycota mitogenomes were classified into different position classes (Pcls) using the *cox1* gene of *G. calidophilum* as the reference. Each Pcl was constituted by introns inserted at the same position of corresponding *cox1* gene and named according to its insertion site in the aligned corresponding reference sequence (nt). The Pcls present in more than 1/5 of Basidiomycota species were considered as common Pcls in Basidiomycota, while introns detected in less than 1/5 of Basidiomycota species were considered to be rare introns. Phylogenetic positions of the 79 Basidiomycota species were established using the Bayesian inference (BI) method and Maximum Likelihood (ML) method based on concatenated mitochondrial genes. Species information is shown in Supplementary Table 6.

We further classified introns of *cox1* genes into different position classes (Pcls). In the present study, we detected 47 Pcls in the *cox1* genes of 79 Basidiomycota species (Figure 6). Pcls present in more than 1/5 of Basidiomycota species were considered as common Pcls in Basidiomycota, while introns detected in less than 1/5 of Basidiomycota species were considered as rare introns. In the present study, 12 common Pcls and 35 rare Pcls were detected in the 79 Basidiomycota species. The P383 was the most common introns in Basidiomycota, which distributed in 40 of the 79 basidiomycete species. P706 and P1107 were also widely distributed in Basidiomycota, which could be detected in 35 and 34 basidiomycete species, respectively. However, 14 Pcls could only be detected in only one of the 79 basidiomycete species, including P166, P193, and P218, P309, P318, etc. A total of 20 Pcls were detected in the 8 Boletales species, of which 10 were common introns in Basidiomycota. The quantity and class of introns in Boletales species varied greatly, indicating intron loss/gain occurred in the evolution of Boletales. The Pcls of the two novel mitogenomes we assembled in this study varied, and P717 present in *P. tinctorius* has not been detected in other Boletales species. However, the novel Pcl (P717) in Boletales was also detected in *Lyophyllum shimeji* (Li et al., 2019b), *Ganoderma calidophilum* (Li et al., 2019c), and *Cryptococcus gattii* (Gomes et al., 2018) from other orders, indicating potential horizontal gene transfer events.

### Comparative Mitogenome Analysis and Phylogenetic Analysis

**FIGURE 7:**
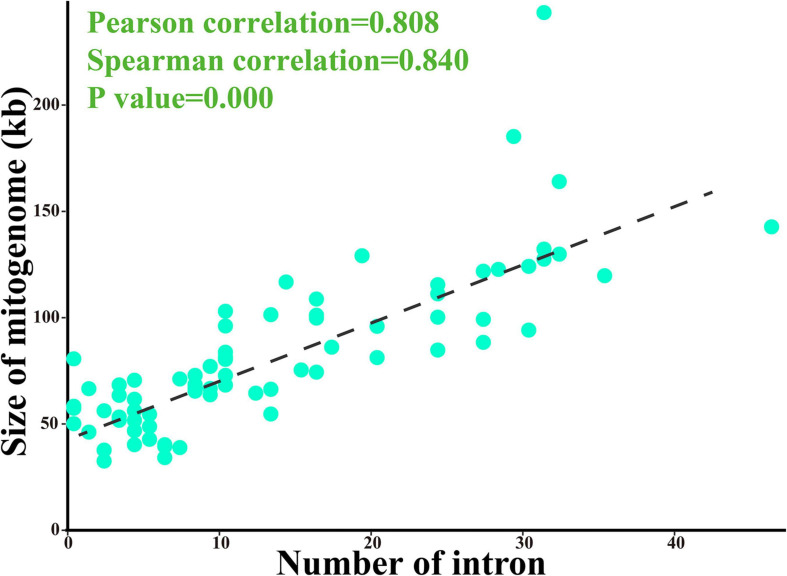
Pearson and spearman correlation analyses of mitogenome size and intron number of 79 basidiomycetes.

Comparative mitogenomic analysis showed that the two *Pisolithus* mitogenomes were smaller than the four mitogenomes from the genera *Coniophora* and *Rhizopogon*, and larger than the two mitogenomes from the genus *Paxillus* (Supplementary Table 1). The average GC content of the two *Pisolithus* mitogenomes (22.76%) was lower that of the two species from the genus *Coniophora*, but larger than that of the other four Boletales species. All the 8 Boletales species contained negative AT skews and positive GC skews. Comparative mitogenome analysis indicated that the number of non-intronic ORFs, plasmid-derived genes, PCGs with unknown functions, introns, intronic ORFs, and tRNA genes varied between Boletales species. All the eight Boletales species contained two rRNA genes. The two *Pisolithus* mitogenomes were found containing the lowest intra-genomic repeats in their mitogenomes than in the other six Boletales species. The proportions of introns, protein coding region, intergenic region, and RNA region were also variable in different Boletales species. The correlation analysis of mitogenome size and intron number of 79 basidiomycetes showed that the mitogenome size had a significant correlation with the number of introns (*p* < 0.01), with the pearson correlation coefficient 0.808 and the spearman correlation coefficient 0.840 (Figure 7).

**FIGURE 8:**
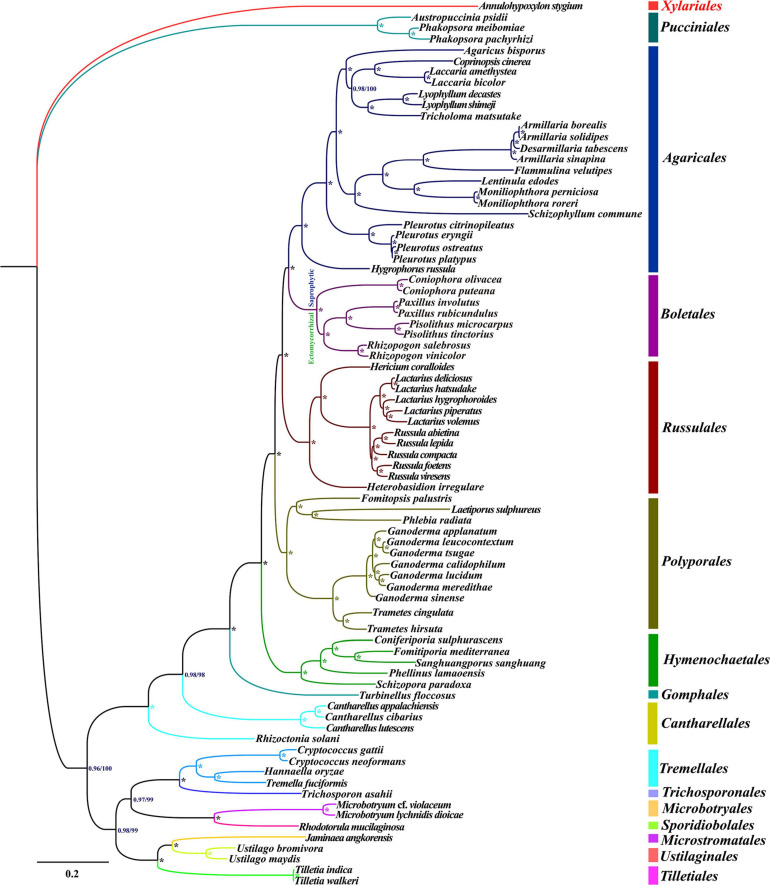
Molecular phylogeny of 79 Basidiomycota species based on Bayesian inference (BI) and Maximum likelihood (ML) analysis of 15 protein coding genes and two rRNA genes. Support values are Bayesian posterior probabilities (BPP, before slash) and bootstrap values (BS, after slash). The asterisk indicates that the BPP value is one and the BS value is 100 of the branch. Species and NCBI accession numbers for mitogenomes used in the phylogenetic analysis are provided in Supplementary Table 6.

An identical and well-supported phylogenetic tree for 79 basidiomycete species was obtained using both Maximum likelihood (ML) and Bayesian inference (BI) methods (Figure 8). The combined mitochondrial gene dataset (15 core PCGs) was used to conduct the phylogenetic analysis. We found all major clades within the phylogenetic tree had good supported values (BPP ≥ 0.96; BS ≥ 98). The 79 basidiomycete species could be divided into 15 major clades in the phylogenetic tree, corresponding to the orders Agaricales, Boletales, Cantharellales, Gomphales, Hymenochaetales, Microbotryales, Microstromatales, Polyporales, Pucciniales, Russulales, Sporidiobolales, Tilletiales, Tremellales, Trichosporonales, and Ustilaginales (Supplementary Table 6). The eight Boletales species could be assigned into two groups, wherein the first group comprised two *Coniophora* species, and the second group comprised six species from several genera, including *Rhizopogon*, *Paxillus*, and *Pisolithus*. The results indicated that the ectomycorrhizal Boletales species may have common origin. The results also showed that the two *Pisolithus* species exhibited a close relationship with *Paxillus* species.

## Discussion

### Variations of Gene Content in Boletales Mitogenomes

It has been reported that the mitogenome of eukaryotes is obtained from bacteria through endosymbiosis (Lang et al., 1999; Munoz-Gomez et al., 2017). In the process of evolution, most of the mitochondrial genes have been transferred to the nuclear genomes (Adams et al., 2002; Adams and Palmer, 2003). However, most animal and fungal mitogenomes retain a whole set of core PCGs in the evolutionary process, which is believed to facilitate the local control of oxidative phosphorylation (Allen, 2015; Bjorkholm et al., 2015). In the present study, we found that these core PCGs varied in length and base composition between different Boletales species. The *rps3* gene differentiated greatly between different Boletales species, while *atp8* and *atp9* genes were quite conservative between Boletales species, indicating that different mitochondrial genes had different evolution rates in Boletales. The *rps3* gene of basidiomycetes is an ancient gene which come from alphaproteobacteria and was relocated to another region of the mitogenome (Korovesi et al., 2018). Previous studies found that the *rps3* gene was probably implicated in the assembly of the mitochondrial small (37S) ribosomal subunit (Seif et al., 2005). In the present study, we found that the *rps3* gene may have been subjected to positive selection between some Boletales species. In addition to core PCGs, we also detected a series of non-conserved PCGs in Boletales species, including plasmid-derived genes, intronic ORFs and PCGs with unknown functions. The number and species of these non-conserved PCGs varied greatly in different Boletales species. Plasmid-derived genes were thought to be obtained from mitochondrial plasmids (Himmelstrand et al., 2014), which were considered as self-replicating genetic elements. Mitochondrial plasmids were free-standing in some Basidiomycota species (Yui et al., 1988; Nakai et al., 2000), and integrated into mitogenomes in some other Basidiomycota species (Formighieri et al., 2008; Ferandon et al., 2013). The dynamic changes of mitochondrial plasmids also play an important role in the evolution of fungal mitogenome (Himmelstrand et al., 2014; Li et al., 2020a; Wang et al., 2020). In addition, different numbers of PCGs with unknown functions have been detected in the eight Boletales mitogenomes. Further studies are needed to fully understand the role of these unknown functional PCGs in species differentiation and ecological adaptation of Boletales. We also detected 7 out of 15 variable sites in the D arm of tRNA genes, which showed that the D arm was highly variable in the two *Pisolithus* mitogenomes.

### Gene Rearrangements in Boletales Mitogenomes

Mitochondrial gene arrangements provide abundant information for understanding the evolutionary status of species (Beaudet et al., 2013; Zhang et al., 2017; Li et al., 2018b). In this study, we found that five out of the eight Boletales species had identical mitochondrial gene arrangement, which may represent the gene arrangement of the ancestors of ectomycorrhizal Boletales species. The identical mitochondrial gene arrangement was found distributed in species from the genera, *Rhizopogon* (Li et al., 2019a), *Paxillus* (Li et al., 2020b), and *Pisoliths*, indicating close phylogenetic relationships between these species. Large-scale gene rearrangements were detected in the two saprophytic Boletales species (*Coniophora*) and the ectomycorrhizal fungus *R. salebrosus*. A total of 4, 14 and 5 out of the 17 genes rearranged in mitogenomes of *C. olivacea*, *C. puteana*, and *R. salebrosus*, respectively, compared with the mitochondrial gene arrangement of putative ectomycorrhizal Boletales ancestors. Up to now, the mechanism of mitochondrial gene rearrangement in fungi has not been fully revealed. Previous studies have shown that the accumulation of repetitive sequences in fungal mitogenomes may contribute to the mitochondrial gene rearrangements in fungi (Aguileta et al., 2014). However, in this study, we found weak correlations between repeat content and gene rearrangement in Boletales. Few repeats were detected in the mitogenome of *R. salebrosus* (Li et al., 2019a), but a large-scale gene rearrangements were found in it. Therefore, there may be other models or mechanisms for Boletales mitochondrial genome rearrangement.

### Introns Dynamics in *cox1* Genes of Basidiomycota Mitogenomes

As mobile genetic elements in fungal mitogenome, intron dynamics play an important role in altering organization or size of fungal mitogenomes (Himmelstrand et al., 2014; Gomes et al., 2018; Fan et al., 2019; Li et al., 2021a). In the present study, we found the number and class of basidiomycete introns varied greatly, even between closely related basidiomycete species, and a significant correlation between the mitogenome size and intron number of 79 basidiomycetes were detected. A total of 1,071 introns were detected in the 79 basidiomycete species, with each species containing 0–46 introns. The introns of basidiomycetes were unevenly distributed in the core PCGs or rRNA genes of basidiomycetes, of which *cox1* gene was the largest host gene of basidiomycete introns. A total of 47 Pcls were detected in the 79 basidiomycete species, and the P383 was the most widely distributed intron class. We found the classes and numbers of introns in the eight Boletales species tested varied greatly, which indicated intron loss/gain events occurred in the evolution of Boletales. Some introns of the Boletales were lost in the evolutionary process, resulting in the relatively small size of Boletales in Basidiomycota. The intron P717 intron was only found in *Pisoliths tinctorius* of the eight Boletales species, while homologous introns were detected in distant species (Li et al., 2019c), indicating potential intron transfer events.

### Reconstruction of Basidiomycete Phylogeny Based on Mitochondrial Gene

Basidiomycetes, the largest mushroom-forming fungal group on the earth, play an important role in ecosystem, industry and medicine (Cahan and Kennell, 2005; Maciel et al., 2010; Martinkova et al., 2016). Understanding the origin, evolutionary and phylogenetic relationships of basidiomycetes will lay a foundation for the effective utilization of basidiomycetes. However, it is difficult to classify or identify basidiomycetes only by morphology, which is mainly due to the changeable and overlapping morphological characteristics or convergent evolution of basidiomycetes (Li et al., 2019b). The introduction of molecular markers promotes the understanding of basidiomycete origin and evolution. Up to now, the rRNA ITS sequence is the most commonly used molecular marker in basidiomycete phylogeny (Schoch et al., 2012). Nuclear genomes of basidiomycetes were also used for phylogeny of basidiomycetes (Cornell et al., 2007; Liu et al., 2017). The mitogenome of basidiomycetes can provide more genetic information than ITS sequence, and it is easier to be obtained than nuclear genome. Therefore, mitogenome of basidiomycetes may become a universal molecular marker to analyze the origin and phylogeny of basidiomycetes (Sandor et al., 2018; Wang et al., 2018; Li et al., 2020c; Wang and Xu, 2020). In the present study, more than 2/3 of basidiomycete mitogenomes available in the public database (see text footnote 1) were included in the phylogenetic study. A well-supported phylogenetic tree for the 79 basidiomycetes was obtained based on mitochondrial gene dataset, which showed that mitochondrial genome is an effective tool to analyze phylogenetic relationship of basidiomycetes. More mitogenomes of basidiomycetes need to be resolved to reconstruct the origin and evolution of basidiomycetes in the future.

## Data Availability Statement

The complete mitogenomes of P. microcarpus and P. tinctorius were deposited in the GenBank database under the accession numbers of MT577034 and MT577035, respectively.

## Author Contributions

MG and QL: conceived and designed experiments. PW, TY, JY, YQ, and YR: analyzed the data. PW and QL: wrote and review the manuscript. All authors contributed to the article and approved the submitted version.

## Conflict of Interest

The authors declare that the research was conducted in the absence of any commercial or financial relationships that could be construed as a potential conflict of interest.

## Footnotes

1 https://www.ncbi.nlm.nih.gov/genome/browse#!/overview/

2 http://www.dnastar.com/
